# First national record of *Quasipaaverrucospinosa* (Bourret, 1937) (Amphibia: Anura: Dicroglossidae) from Thailand with further comment on its taxonomic status

**DOI:** 10.3897/BDJ.9.e70473

**Published:** 2021-09-30

**Authors:** Chatmongkon Suwannapoom, Tan Van Nguyen, Nikolay A. Poyarkov, Yun-He Wu, Parinya Pawangkhanant, Sengvilay Lorphengsy, Jing Che

**Affiliations:** 1 Division of Fishery, School of Agriculture and Natural Resources, University of Phayao, Phayao, Thailand Division of Fishery, School of Agriculture and Natural Resources, University of Phayao Phayao Thailand; 2 Department of Species Conservation, Save Vietnam’s Wildlife, Ninh Binh, Vietnam Department of Species Conservation, Save Vietnam’s Wildlife Ninh Binh Vietnam; 3 Faculty of Biology, Department of Vertebrate Zoology, Moscow State University, Moscow, Russia Faculty of Biology, Department of Vertebrate Zoology, Moscow State University Moscow Russia; 4 Laboratory of Tropical Ecology, Joint Russian-Vietnamese Tropical Research and Technological Center, Hanoi, Vietnam Laboratory of Tropical Ecology, Joint Russian-Vietnamese Tropical Research and Technological Center Hanoi Vietnam; 5 State Key Laboratory of Genetic Resources and Evolution, Kunming Institute of Zoology, Chinese Academy of Sciences, Kunming, Yunnan, China State Key Laboratory of Genetic Resources and Evolution, Kunming Institute of Zoology, Chinese Academy of Sciences, Kunming Yunnan China; 6 Division of Biotechnology, School of Agriculture and Natural Resources, University of Phayao, Phayao, Thailand Division of Biotechnology, School of Agriculture and Natural Resources, University of Phayao Phayao Thailand

**Keywords:** *Quasipaaverrucospinosa*, new record, 16S rRNA, morphology, Nan Province

## Abstract

**Background:**

Spiny Frog *Quasipaa* is a genus of frogs that belongs to a relatively poorly known group. Most of the species distribution has been recorded in China; however, a few incidences of identification have occurred in the eastern part of Indochina. To date, only one species (*Quasipaafasciculispina*) of *Quasipaa* has been recorded from Chanthaburi and Trat Provinces in south-eastern Thailand.

**New information:**

Based on recent fieldwork conducted in northern Thailand, we report a new record of *Quasipaaverrucospinosa* from Doi Phu Kha National Park, Nan Province at an altitude of 900–1000 m a.s.l. Our study has demonstrated that populations of this species are paraphyletic and has revealed deep genetic differences. Therefore, it is recommended that a comprehensive study be undertaken to clarify the taxonomic and geographic distribution of this species for its suitable protection and conservation.

## Introduction

The family Dicroglossidae is a diverse group of amphibians in Thailand. As a direct result of the increasing amounts of attention and effort being devoted to herpetological research studies, the species diversity of this family in Thailand has remarkably increased from 31 species in the year 2011, to 38 species at present (Chuaynkern and Chuaynkern 2012, Poyarkov et al. 2021, Suwannapoom et al. 2021). Most species of frogs of the genus *Quasipaa* are endemic to China, but a few of them have been reported in the eastern part of the Indochinese Peninsula (Vietnam, Laos, Cambodia and Thailand). To date, official records are comprised of 12 recognised species of this genus, of which only one species, namely *Q.fasciculispina*, was reported from south-eastern Thailand (Frost 2021). The Granular Spiny Frog *Quasipaaverrucospinosa* (considered to be at the ‘Near Threatened’ level on the IUCN Red List) was first described by René Bourret in 1937 and was collected from Chapa and Fansipan (now Sa Pa) in Lao Cai Province and Tam Dao National Park in Vinh Phuc Province, northern Vietnam. Currently, the distribution of this species ranges from northern and central Vietnam; southern China; and northwest Laos (Nguyen et al. 2020, Frost 2021).

Recent field surveys, conducted in northern Thailand from 2018 to 2019, revealed a *Quasipaa* species. Morphological and molecular examination indicates that this is closely related to *Q.verrucospinosa* which we report here as a new record to Thailand. Herein, we have reported new records of *Quasipaaverrucospinosa* from Nan Province. In addition, due to incidences of high morphological similarities and a lack of significant research efforts, the taxonomic status of this species remains unclear and overlooked. Based on the results of our molecular analysis, we have determined that there are genetic differences amongst populations of this species. This evidence suggests that this genus contains a number of cryptic species. Consequently, more in-depth research on this genus will be needed in the future.

## Materials and methods

### Sampling

**Figure 1.**
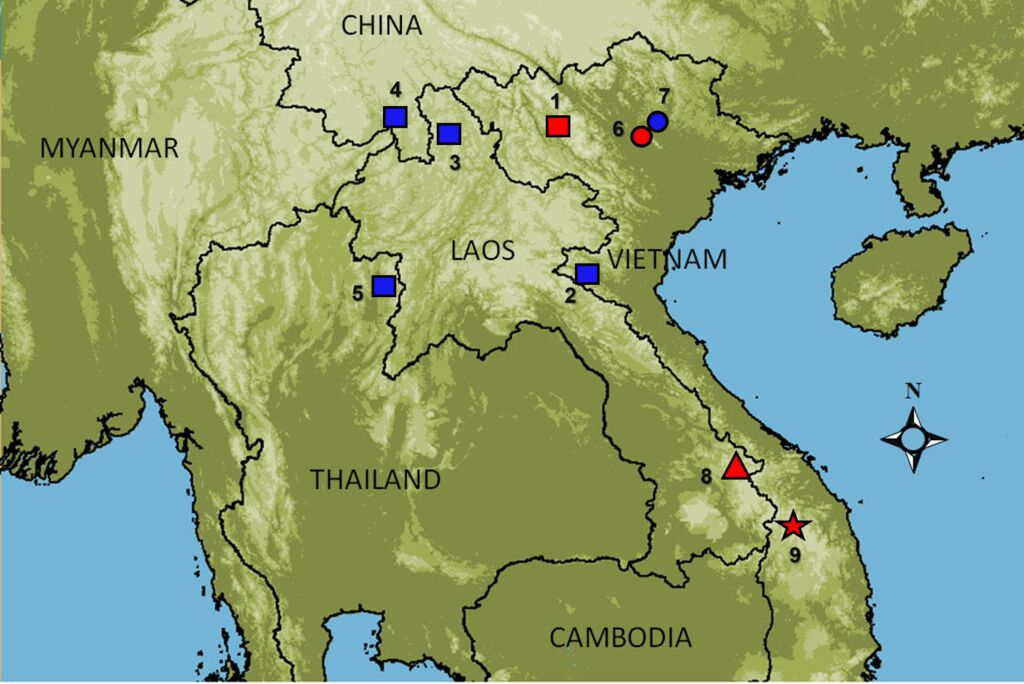
Map showing distribution of species *Quasipaaverrucospinosa* complex. (Red colour: type locality). Localites. *Q.verrucospinosa* sensu stricto; (1) Sapa, Lao Cai, Vietnam; (2) Pu Mat NP., Con Cuong, Nghe An, Vietnam; (3) Phongsaly, Laos; (4) Mengsong, Jinghong, Yunnan, China; (5) Doi Phu Kha National Park, Nan Province, Thailand; Q.cf.verrucospinosa 1; (6) Tam Dao NP., Vinh Phuc, Vietnam; (7) Thai Nguyen, Vietnam; Q.cf.verrucospinosa 2; (8) Kaleum, Xekong, Laos; Q.cf.verrucospinosa 3; (9) Ngoc Linh Mt., Kon Tum, Vietnam.

Field surveys were conducted from October, 2018 to December, 2019 in Doi Phu Kha National Park, Nan Province, Thailand (Fig. 1). A total of 10 specimens were collected and photographed before being euthanised using a 20% solution of benzocaine. The specimens were then fixed and stored in 80% ethanol. Tissue samples were taken for the purpose of genetic analysis prior to preservation of the specimens. The samples were stored in 95% ethanol. Specimens and tissues were subsequently deposited with the herpetological collection of the School of Agriculture and Natural Resources, University of Phayao (AUP), Phayao, Thailand.

### Morphological characteristics

Measurements were taken with a digital caliper to the nearest 0.1 mm. Abbreviations are as follows Matsui (1984): Snout-vent length (SVL); Head length (HL); Snout-length (SL); Eye length (EL); Nostril-eyelid length (N-EL); Head width (HW); Internarial distance (IND); Interorbital distance (IOD); Upper eyelid width (UEW); Forelimbs length (FLL); Lower arm length (LAL); Hand length (HAL); First finger length (1FL), Inner palmar tubercle length (IPTL); Outer palmar tubercle length (OPTL); Third finger disc diameter (3FDD); Hindlimb length (HLL); Tibia length (TL); Foot length (FL) Inner metatarsal tubercle length (IMTL); (1TOEL); fourth toe disc diameter (4TDD); third finger disc diameter (3FDD); Outer metatarsal tubercle length (OMTL). Sex was determined by the presence of nuptial pads and gonadal inspection. Other abbreviations: Mt.: Mountain; NR.: Nature reserve; NP.: National Park; a.s.l.: above sea level.

### Molecular analysis

Genomic DNA was extracted from liver tissues that had been preserved in 95% ethanol using the standard phenol-chloroform extraction protocol (Sambrook et al. 1989). Partial fragments of the mitochondrial 16S rRNA were amplified for all samples via the polymerase chain reaction (PCR) using the following primers: 16SAR (5'-CGCCTGTTTAYCAAAAACAT-3') and 16SBR (5'-CCGGTYTGAACTCAGATCAYGT-3'; Kocher et al. 1989). PCR amplifications were performed in a 25 µl reaction volume with the following cycling conditions: an initial denaturing step at 95°C for 4 min, 35 cycles of denaturing at 94°C for 40 s, annealing at 55°C for 30 s, an extension step at 72°C for 1 min and a final extension step at 72°C for 10 min. Sequence analysis was performed on an ABI PRISM 3730xl DNA sequencer (Applied Biosystems). Newly-obtained sequences were deposited in GenBank under the accession numbers OK178934-OK178940 (for more details, see Suppl. material 1).

### Phylogenetic analyses

To estimate the matrilineal genealogy of the genus *Quasipaa*, newly-obtained 16S rRNA sequences were used together with the previously-published sequences of *Quasipaaverrucospinosa*, as well as representative sequences of 44 other species of *Quasipaa*. Furthermore, we included the five sequences of *Hoplobatrachusrugulosus* (Wiegmann) and *Fejervaryalimnocharis* (Gravenhorst) which were used as the outgroup (see Suppl. material 1). In total, 16S rRNA sequences for the *Quasipaa* specimens were included in the final analysis. The final analysis also included sequences of the *Quasipaaverrucospinosa* species complex obtained from Vietnam, China, Laos and Thailand.

Trees were reconstructed using Bayesian Inference (BI) and Maximum Likelihood (ML). JMODELTEST v.2.1.7 (Darriba et al. 2012) was used to select an appropriate nucleotide substitution model for BI. The GTR+G model was chosen as the best-fit model following the Bayesian information criterion (Posada 2008). The CIPRES web server was selected to implement BI. The Monte Carlo Markov chain length was run for 10,000,000 generations and sampled every 1,000 generations. A burn-in value of 25% was used. Convergence was assessed by the average standard deviation of the split frequencies (below 0.01) and the ESS values (over 200) in TRACER v.1.5 (Rambaut and Drummond 2007). ML was performed using RAxML with 1,000 bootstrap replications (Stamatakis et al. 2008). Mean genetic distances were calculated using uncorrected p-distances established in MEGA v.6.0.6.

## Taxon treatments

### Quasipaa verrucospinosa

(Bourret, 1937)

65BF7EC1-909C-58BE-AE2D-9A7B3157EDF3

#### Materials

- **Type status:** Other material. **Occurrence:** catalogNumber: AUP-00392; individualCount: 1; sex: female; lifeStage: adult; **Taxon:** scientificName: *Quasipaaverrucospinosa*; class: Amphibia; order: Anura; family: Dicroglossidae; genus: Quasipaa; specificEpithet: *verrucospinosa*; **Location:** country: Thailand; countryCode: TL; stateProvince: Nan; locality: Doi Phu Kha; verbatimElevation: 900–1000; verbatimLatitude: 21°19.66'N; verbatimLongitude: 103°36.26'E; verbatimCoordinateSystem: WGS84; **Event:** eventRemarks: collected by C. Suwannapoom, P. Pawangkhanant; **Record Level:** basisOfRecord: preserved specime
- **Type status:** Other material. **Occurrence:** catalogNumber: AUP-00393; individualCount: 1; sex: female; lifeStage: adult; **Taxon:** scientificName: *Quasipaaverrucospinosa*; **Record Level:** basisOfRecord: preserved specimen; dynamicProperties: collection date, collector and location as the AUP-00392
- **Type status:** Other material. **Occurrence:** catalogNumber: AUP-00530; individualCount: 1; sex: female; lifeStage: adult; **Taxon:** scientificName: *Quasipaaverrucospinosa*; **Record Level:** basisOfRecord: preserved specimen; dynamicProperties: collection date, collector and location as the AUP-00392
- **Type status:** Other material. **Occurrence:** catalogNumber: AUP-00531; individualCount: 1; sex: male; lifeStage: adult; **Taxon:** scientificName: *Quasipaaverrucospinosa*; **Record Level:** basisOfRecord: preserved specimen; dynamicProperties: collection date, collector and location as the AUP-00392
- **Type status:** Other material. **Occurrence:** catalogNumber: AUP-00532; individualCount: 1; sex: male; lifeStage: adult; **Taxon:** scientificName: *Quasipaaverrucospinosa*; **Record Level:** basisOfRecord: preserved specimen; dynamicProperties: collection date, collector and location as the AUP-00392
- **Type status:** Other material. **Occurrence:** catalogNumber: AUP-00533; individualCount: 1; sex: male; lifeStage: adult; **Taxon:** scientificName: *Quasipaaverrucospinosa*; **Record Level:** basisOfRecord: preserved specimen; dynamicProperties: collection date, collector and location as the AUP-00392
- **Type status:** Other material. **Occurrence:** catalogNumber: AUP-00534; individualCount: 1; sex: male; lifeStage: adult; **Taxon:** scientificName: *Quasipaaverrucospinosa*; **Record Level:** basisOfRecord: preserved specimen; dynamicProperties: collection date, collector and location as the AUP-00392
- **Type status:** Other material. **Occurrence:** catalogNumber: AUP-01609; individualCount: 1; sex: female; lifeStage: adult; **Taxon:** scientificName: *Quasipaaverrucospinosa*; **Record Level:** basisOfRecord: preserved specimen; dynamicProperties: collection date, collector and location as the AUP-00392

#### Description

Morphological characteristics of the specimens (n = 10) obtained from Nan Province agreed with the descriptions of Bourret (1937), Fei et al. (2010), Nguyen et al. (2020): SVL 99.1–116.8 mm in males (n = 4) and 83.4–116.6 mm in females (n = 6). Other morphological measurements of all specimens collected from Nan Province are presented in Suppl. material 3. Body rather stout; head wider than long; snout round, slightly protruding, longer than horizontal diameter of eye; canthus rostralis distinct; loreal region oblique, moderately concave; nostril lateral, oval with lateral flap of skin, closer to tip of snout than to eye; interorbital broader than upper eyelid and internarial distance; eyes large, pupils oval; tympanum small, distinct; supratympanic fold present; vomerine teeth present; tongue heart-shaped, notched posteriorly; vocal openings absent. Forelimbs long, robust; relative finger lengths II < I < IV < III; fingers without dermal fringe, free of webbing; tips of fingers, slightly enlarged, without discs; subarticular tubercle developed; palmar tubercles two, oval; nuptial pads spines present. Hindlimbs short, strong; thigh longer than tibia; tips of toes rounded, distinctly enlarged, without discs; relative toes lengths I < II < V < III < IV; toe webbing complete; subarticular tubercles prominent, rounded; inner metatarsal tubercle distinct, shorter than length of toe I.

**Figure 2.**
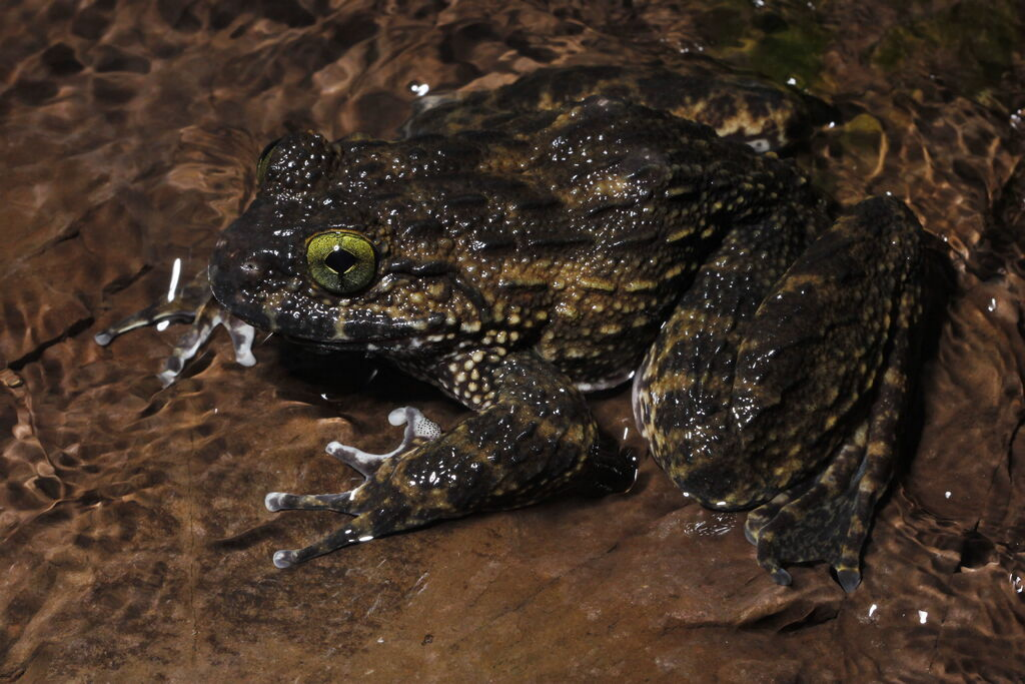
*Quasipaaverrucospinosa*, male, in situ from Nan Province, Thailand. Photo by P. Pawangkhanant.

**Figure 3.**
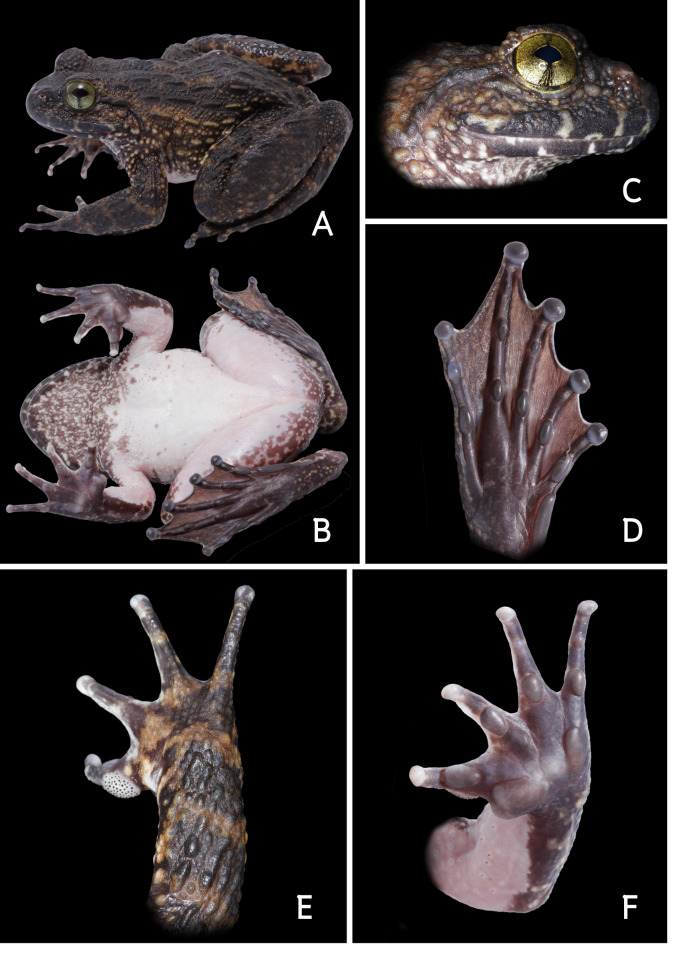
*Quasipaaverrucospinosa* (AUP-00393), male. **A.** Dorsal view; **B.** Ventral view; **C.** Lateral view of head; **D.** Thenar view of the left foot; **E.** Palmar view of the right hand; **F.** Thenar view of the left hand.

Skin. Dorsal surface with very rough back covered by short, thick, ridges and round tubercles. Sides covered by oval tubercles with dark spines; ventral surface smooth. During the breeding season (May to October), males have black spikes on finger I, chin, chest and underarms. Colouration of dorsum is grey-brown with dark brown spots; ventral surface cream-coloured, except for the chin with dark markings; iris dark green (Figs 2, 3).

#### Distribution

This species is known to be from northern and central Vietnam, southern China and northwest Laos (Nguyen et al. 2020). This is the first record of this species in Thailand, ca. 440 km southwest from the type locality in Sa Pa District, Lao Cai Province, Vietnam (Bourret 1937) (Fig. 1).

#### Ecology

**Figure 4.**
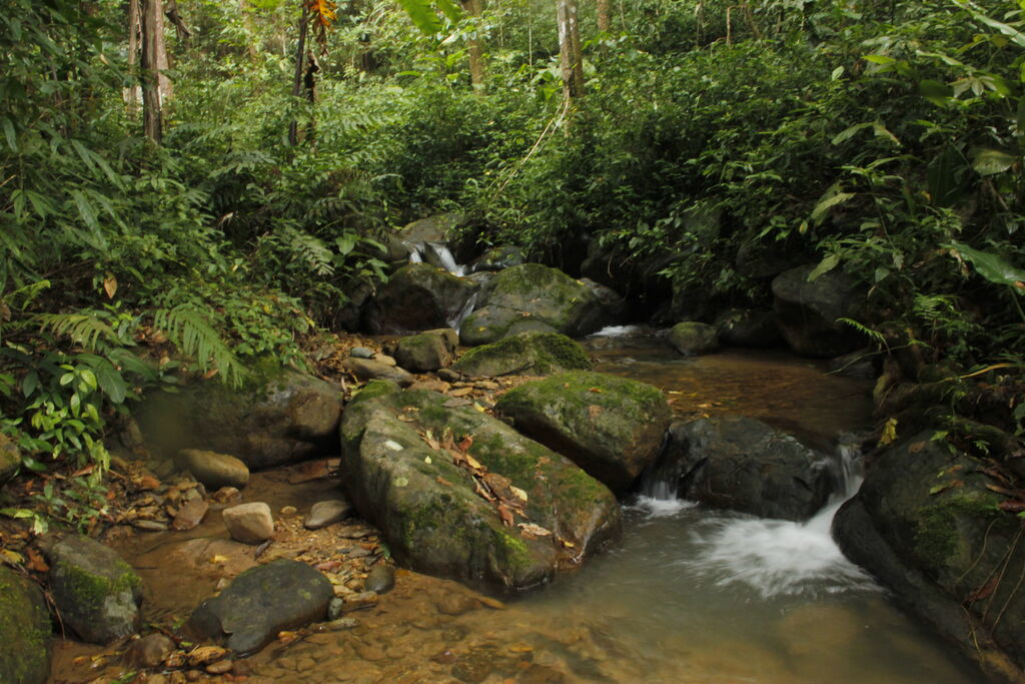
Habitat of *Q.verrucospinosa* at Doi Phu Ka National Park, Nan Province, Thailand. Photo by P. Pawangkhanant.

Individual specimens collected from Doi Phu Kha National Park were found at night between 19:00 and 23:00 h on both small and large rocks in medium-sized streams and in forests at elevations between 900–1000 m a.s.l. The surrounding habitat comprised secondary evergreen forests of medium growth. Other amphibian species found to be sympatric include: *Limnonectestaylori* Matsui, Panha, Khonsue & Kuraishi and *L.bannaensis* Ye, Fei, Xie & Jiang. Advertisement calls of the males can be heard within or beside rocky streams from May to October (Fig. 4).

## Analysis

### Molecular phylogeny

**Figure 5.**
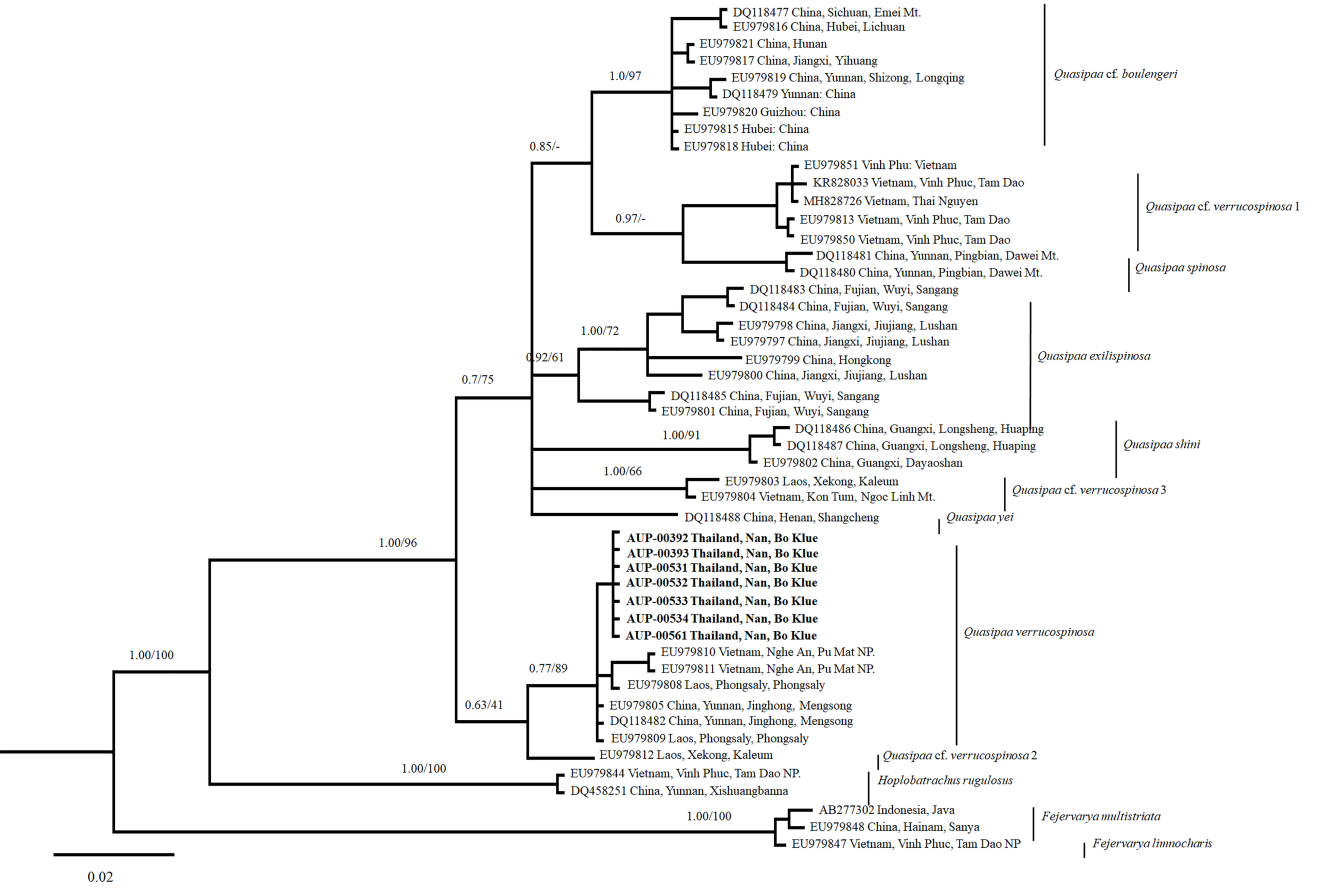
Maximum Likelihood consensus tree of the *Quasipaaverrucospinosa* group, based on the 16S rRNA gene.

Sequencing generated a total of ~ 550 base pairs (bp) of 16S rRNA data for *Quasipaaverrucospinosa*. Interspecific uncorrected *p*-distances were recorded between the newly-discovered population of *Q.verrucospinosa* collected from Bo Klue, Doi Phu Kha National Park, Nan Province in Thailand, while the other known species of *Quasipaa* varied from 2.7% (with Q.cf.verrucospinosa 2) to 7.1% (with *Q.shini*) (Suppl. material 2). The uncorrected *p*-distance value between the newly-found populations of *Q.verrucospinosa*, obtained from Nan Province and topotypic *Q.verrucospinosa* populations (Pu Mat NP., Nghe An Province, Vietnam; Phongsaly Province, Laos and Jinghong, Yunnan Province, China), was 0.2%. Furthermore, both ML and BI analyses placed the Nan population in one clade along with topotypic *Q.verrucospinosa* in conjunction with strong node support (Fig. 5).

## Discussion

Due to high morphological similarities, the species of the genus *Quasipaa* are distinguished from each other mainly by the pattern and number of genital spikes in the areas of the first finger, chest, chin and underarms of males during their breeding season. Therefore, it can be considerably difficult for herpetologists to identify this species. Consequently, the taxonomic status of the species in this group is both controversial and unclear. Additionally, there has been a notable lack of interest in and integrated research (morphological and molecular approach) conducted on this species. However, this species has been determined to be especially widespread (e.g. *Quasipaaverrucospinosa* sensu lato discussed here previously has been misidentified and placed under the names *Chaparanadelacouri* (currently *Quasipaadelacouri*, restricted in northern Vietnam); *Paaboulengeri* (currently *Quasipaaboulengeri* ,restricted in south-eastern and south-western in China); *Paaspinosa* (currently *Quasipaaspinosa*, restriced in south-eastern China and probably in northern Vietnam) and *Paa* spp. (see Che et al. 2009, Che et al. 2010, Yan et al. 2021). For all of these reasons, a high degree of complexity has been associated with the accurate identification and classification of this species.

*Quasipaaverrucospinosa* has originally been described, based on specimens collected throughout northern Vietnam from Sapa (Lao Cai Province) and Tam Dao NP. (Vinh Phuc Province). These two locations are situated on opposite sides of the Red River. Dubois (1987) has designated the MNNH 1948.132 as the lectotype of this species and restricted the type locality in Sapa, Lao Cai. Unfortunately, we have not had access to available DNA sequences for samples collected from Sa Pa, Lao Cai Province (as this was beyond the scope of this study). Moreover, our molecular data have demonstrated that there are significant genetic differences amongst the samples of this species. Specifically, this species is classified into four groups as follows: group A: encompasses samples obtained from the northwest section of the Red River [from Nghe An (northwest Vietnam), Phongsaly (northern Laos); Yunnan (northwest China) and Nan (northern Thailand)]; group B: encompasses samples obtained from the north-eastern section of the Red River (from Vinh Phuc and Thai Nguyen Provinces, northeast Vietnam); group C: includes specimens collected form Xekong Province (south Laos) and group D: includes samples collected from Kon Tum Province (central Vietnam) and Xekong Province (south Laos) (see Fig. 5). As has been mentioned above, noteworthy differences have been observed in the genetic distances amongst the distribution areas of this species. The Red River has been identified as a geographical barrier for many amphibian species that display similar morphology (see Bain and Hurley 2011, Bain et al. 2009, Lyu et al. 2020, Yuan et al. 2016). On the other hand, based on examinations of the morphological characteristics of the specimens of this species collected from different areas of Vietnam (including the locations mentioned in our molecular study), we noticed that there are differences in the patterns and numbers of black spines in the area of the first finger, chin, throat and underarm of the specimens, as well as in the colour of their eyes (iris dark green in northwest of Red River vs. dark brown in northeast of Red River (T.V. Nguyen, per. obs.). Consequently, we recognise Sapa, Lao Cai Province as the true type locality of this species. Thus, the specimens obtained from group A should be assigned to *Q.verrucospinosa* sensu stricto. Specimens previously placed in other groups should be assigned to groups Q.cf.verrucospinosa in order to more accurately correspond with specimens in groups B, C and D (Fig. 5). Therefore, a comprehensive assessment of the taxonomic status of this species should be prioritised. Thus, a degree of urgency should be applied to determine the true distribution area of this species, as well as to the establishment of a range of effective conservation measures.

## Supplementary Material

XML Treatment for Quasipaa verrucospinosa

4A2F8312-9787-56D5-BA9B-24B6E33D9443 10.3897/BDJ.9.e70473.suppl1 Supplementary material 1 Sequences and voucher specimens of *Quasipaa* and outgroup taxa used in molecular analyses for this study. For sampling localities, see Fig. 1. (Notes: NP= National Park; NR=Natural Reserve; Mt.= Mountain; N/a: Not available). Data type Table of sequences and voucher specimens of Quasipaa and outgroup taxa used in molecular analyses File: oo_592305.docx https://binary.pensoft.net/file/592305 Suwannapoom et al.

FECF5CBD-031A-5187-912F-4A426C20EA22 10.3897/BDJ.9.e70473.suppl2 Supplementary material 2 The pairwise uncorrected p-distance (%) of 16S rRNA gene between species of *Quasipaa*. Data type p-distance File: oo_554712.docx https://binary.pensoft.net/file/554712 Suwannapoom et al.

4B07CD53-2032-5B61-904D-4E72DA05A73C 10.3897/BDJ.9.e70473.suppl3 Supplementary material 3 Measurement (in mm) and proportions of the series of *Quasipaaverrucospinosa* in Thailand. (M= Male, F= Female; for other abbreviations see Materials and methods). Data type Measurement of Quasipaa verrucospinosa in Thailand File: oo_554755.docx https://binary.pensoft.net/file/554755 Suwannapoom et al.
