# Supplementary material for: First national record of Quasipaaverrucospinosa (Bourret, 1937) (Amphibia: Anura: Dicroglossidae) from Thailand with further comment on its taxonomic status
Source: Biodivers Data J. 2021 Sep 30;9:e70473. doi: 10.3897/BDJ.9.e70473 (PMC8497459; doi:10.3897/BDJ.9.e70473)
Supplement: Supplementary material 1 — Sequences and voucher specimens of Quasipaa and outgroup taxa used in molecular analyses for this study. For sampling localities, see Fig. 1. (Notes: NP= National Park; NR=Natural Reserve; Mt.= Mountain; N/a: Not available). [file bdj-09-e70473-s001.docx]

Table 1. Sequences and voucher specimens of *Quasipaa* and outgroup taxa used in molecular analyses for this study. For sampling localities see Fig. 1. (**Notes**: NP= National Park; NR=Natural Reserve; Mt.= Mountain; N/a: Not available).

| # | Species identification | | Locality | GenBank No. | Specimen ID | Reference |
| --- | --- | --- | --- | --- | --- | --- |
|  | Previous study | This study |  |  |  |  |
| 1 | *Paa boulengeri* 1 | *Quasipaa boulengeri* | China, Sichuan, Emei Mt. | DQ118477 | SCUM 37989 | Chen et al. 2009 |
| 2 | *Paa boulengeri* 2 | *Quasipaa boulengeri* | China, Hunan | EU979821 | YNU-HUHU 01 | Chen et al. 2009 |
| 3 | *Paa boulengeri* 3 | *Quasipaa boulengeri* | China, Guizhou, Maolan NR. | EU979820 | YNU-HU2003061301 | Chen et al. 2009 |
| 4 | *Paa boulengeri* 4 | *Quasipaa boulengeri* | China, Yunnan, Shizong, Longqing | EU979819 | YNU-HU20024060 | Chen et al. 2009 |
| 5 | *Paa boulengeri* 5 | *Quasipaa boulengeri* | China, Yunnan, Shizong, Longqing | DQ118479 | YNU-HU20024061 | Chen et al. 2009 |
| 6 | *Paa boulengeri* 7 | *Quasipaa boulengeri* | China, Jiangxi, Yihuang | EU979817 | KIZ-JX246 | Chen et al. 2009 |
| 7 | *Paa boulengeri* 8 | *Quasipaa boulengeri* | China, Hubei, Yingchang | EU979815 | KIZ-HUB292 | Chen et al. 2009 |
| 8 | *Paa boulengeri* 9 | *Quasipaa boulengeri* | China, Hubei, Yingchang | EU979818 | KIZ-HUB293 | Chen et al. 2009 |
| 9 | *Paa boulengeri* 10 | *Quasipaa boulengeri* | China, Hubei, Lichuan | EU979816 | KIZ-HUB274 | Chen et al. 2009 |
| 10 | *Paa exilispinosa* 1 | *Quasipaa exilispinosa* | China, Fujian, Wuyi, Sangang | DQ118484 | YNU-HU20026023 | Chen et al. 2009 |
| 11 | *Paa exilispinosa* 2 | *Quasipaa exilispinosa* | China, Fujian, Wuyi, Sangang | DQ118483 | YNU-HU20026022 | Chen et al. 2009 |
| 12 | *Paa exilispinosa* 3 | *Quasipaa exilispinosa* | China, Hongkong | EU979799 | MVZ230391 | Chen et al. 2009 |
| 13 | *Paa spinosa* 5 | *Quasipaa exilispinosa* | China, Jiangxi, Jiujiang, Lushan | EU979798 | KIZ-JX0709001 | Chen et al. 2009 |
| 14 | *Paa spinosa* 6 | *Quasipaa exilispinosa* | China, Jiangxi, Jiujiang, Lushan | EU979797 | KIZ-JX0709002 | Chen et al. 2009 |
| 15 | *Paa spinosa* 7 | *Quasipaa exilispinosa* | China, Jiangxi, Jiujiang, Lushan | EU979800 | KIZ-C21 | Chen et al. 2009 |
| 16 | *Paa jiulongensis* 1 | *Quasipaa jiulongensis* | China, Fujian, Wuyi, Sangang | DQ118485 | YNU-HU200206036 | Chen et al. 2009 |
| 17 | *Paa jiulongensis* 2 | *Quasipaa jiulongensis* | China, Fujian, Wuyi, Sangang | EU979801 | YNU-HU200206037 | Chen et al. 2009 |
| 18 | *Paa shini* 1 | *Quasipaa shini* | China, Guangxi, Longsheng, Huaping | DQ118486 | YNU-HU20025002 | Chen et al. 2009 |
| 19 | *Paa shini* 2 | *Quasipaa shini* | China, Guangxi, Longsheng, Huaping | DQ118487 | YNU-HU20025001 | Chen et al. 2009 |
| 20 | *Paa shini* 3 | *Quasipaa shini* | China, Guangxi, Dayaoshan | EU979802 | SCUM 060702L | Chen et al. 2009 |
| 21 | *Paa spinosa* 1 | *Quasipaa spinosa* | China, Yunnan, Pingbian, Dawei Mt. | DQ118480 | YNU-HU20024040 | Chen et al. 2009 |
| 22 | *Paa spinosa* 2 | *Quasipaa spinosa* | China, Yunnan, Pingbian, Dawei Mt. | DQ118481 | YNU-HU20024042 | Chen et al. 2009 |
| 23 | N/a | *Quasipaa verrucospinosa* | Thailand, Nan, Bo Klue, Doi Phu Kha NP. | **OK178934** | **AUP-00392** | **This study** |
| 24 | N/a | *Quasipaa verrucospinosa* | Thailand, Nan, Bo Klue, Doi Phu Kha NP. | **OK178935** | **AUP-00393** | **This study** |
| 25 | N/a | *Quasipaa verrucospinosa* | Thailand, Nan, Bo Klue, Doi Phu Kha NP. | **OK178936** | **AUP-00531** | **This study** |
| 26 | N/a | *Quasipaa verrucospinosa* | Thailand, Nan, Bo Klue, Doi Phu Kha NP. | **OK178937** | **AUP-00532** | **This study** |
| 27 | N/a | *Quasipaa verrucospinosa* | Thailand, Nan, Bo Klue, Doi Phu Kha NP. | **OK178938** | **AUP-00533** | **This study** |
| 28 | N/a | *Quasipaa verrucospinosa* | Thailand, Nan, Bo Klue, Doi Phu Kha NP. | **OK178939** | **AUP-00534** | **This study** |
| 29 | N/a | *Quasipaa verrucospinosa* | Thailand, Nan, Bo Klue, Doi Phu Kha NP. | **OK178940** | **AUP-00561** | **This study** |
| 30 | *Paa verrucospinosa* 1 | *Quasipaa verrucospinosa* | China, Yunnan, Jinghong, Mengsong | EU979805 | YNU-HU 20030724006 | Chen et al. 2009 |
| 31 | *Paa verrucospinosa* 2 | *Quasipaa verrucospinosa* | China, Yunnan, Jinghong, Mengsong | DQ118482 | YNU-HU 20030724005 | Chen et al. 2009 |
| 32 | *Paa* cf. *spinosa* 8 | *Quasipaa verrucospinosa* | Vietnam, Nghe An, Pu Mat NP. | EU979811 | ROM 35181 | Chen et al. 2009 |
| 33 | *Chaparana* cf. *delacouri* 2 | *Quasipaa verrucospinosa* | Vietnam, Nghe An, Pu Mat NP. | EU979810 | FMNH 255623 | Chen et al. 2009 |
| 34 | *Chaparana* cf. *delacouri* 3 | *Quasipaa verrucospinosa* | Laos, Phongsaly, Phongsaly | EU979808 | FMNH 258628 | Chen et al. 2009 |
| 35 | *Paa* sp. | *Quasipaa verrucospinosa* | Laos, Phongsaly, Phongsaly | EU979809 | FMNH 258389 | Chen et al. 2009 |
| 36 | *Paa* cf. *boulengeri* 6 | *Quasipaa* cf. *verrucospinosa* 1 | Vietnam, Vinh Phuc, Tam Dao | EU979851 | MVZ 226340 | Chen et al. 2009 |
| 37 | *Paa verrucospinosa* 3 | *Quasipaa* cf. *verrucospinosa* 1 | Vietnam, Vinh Phuc, Tam Dao | EU979813 | MVZ 223858 | Chen et al. 2009 |
| 38 | *Paa verrucospinosa* 4 | *Quasipaa* cf. *verrucospinosa* 1 | Vietnam, Vinh Phuc, Tam Dao | EU979850 | MVZ 223934 | Chen et al. 2009 |
| 39 | *Quasipaa verrucospinosa* | *Quasipaa* cf. *verrucospinosa* 1 | Vietnam, Vinh Phuc, Tam Dao | KR828033 | K732 | Grosjean et al. 2015 |
| 40 | *Quasipaa verrucospinosa* | *Quasipaa* cf. *verrucospinosa* 1 | Vietnam, Thai Nguyen | MH828726 | TN3 | unpublished |
| 41 | *Chaparana* cf. *delacouri* 1 | *Quasipaa* cf. *verrucospinosa* 2 | Laos, Xekong, Kaleum | EU979812 | FMNH 258619 | Chen et al. 2009 |
| 42 | *Paa* sp. | *Quasipaa* cf. *verrucospinosa* 3 | Laos, Xekong, Kaleum | EU979803 | FMNH 258383 | Chen et al. 2009 |
| 43 | *Paa* sp. | *Quasipaa* cf. *verrucospinosa* 3 | Vietnam, Kon Tum, Ngoc Linh Mt. | EU979804 | ROM 37390 | Chen et al. 2009 |
| 44 | *Quasipaa yei* | *Quasipaa yei* | China, Henan, Shangcheng | DQ118488 | YNU-HU200205151 | Chen et al. 2009 |
| 45 | **Outgroups** |  |  | Outgroups |  |  |
| 46 | *Hoplobatrachus rugulosus* | *Hoplobatrachus rugulosus* | China, Yunnan, Xishuangbanna | DQ458251 | SCUM 0437941 | Chen et al. 2009 |
|  | *Hoplobatrachus rugulosus* | *Hoplobatrachus rugulosus* | Vietnam, Vinh Phuc, Tam Dao NP. | EU979844 | MVZ 224079 | Chen et al. 2009 |
| 47 | *Fejervarya limnocharis* | *Fejervarya multistriata* | Vietnam, Vinh Phuc, Tam Dao NP. | EU979847 | MVZ 226347 | Chen et al. 2009 |
| 48 | *Fejervarya limnocharis* | *Fejervarya multistriata* | China, Hainam, Sanya | EU979848 | SCUM H003CJ | Chen et al. 2009 |
| 49 | *Fejervarya limnocharis* | *Fejervarya limnocharis* | Indonesia, Java | AB277302 | N/a | Kotaki et al. 2008 |
