# Supplementary material for: First national record of Quasipaaverrucospinosa (Bourret, 1937) (Amphibia: Anura: Dicroglossidae) from Thailand with further comment on its taxonomic status
Source: Biodivers Data J. 2021 Sep 30;9:e70473. doi: 10.3897/BDJ.9.e70473 (PMC8497459; doi:10.3897/BDJ.9.e70473)
Supplement: Supplementary material 2 — The pairwise uncorrected p-distance (%) of 16S rRNA gene between species of Quasipaa. [file bdj-09-e70473-s002.docx]

Table 2. The pairwise uncorrected p-distance (%) of 16S rRNA gene between species of *Quasipaa*.

|  | **Species** | 1 | 2 | 3 | 4 | 5 | 6 | 7 | 8 | 9 | 10 | 11 | 12 | 13 | 14 | 15 | 16 | 17 | 18 | 19 | 20 | 21 |
| --- | --- | --- | --- | --- | --- | --- | --- | --- | --- | --- | --- | --- | --- | --- | --- | --- | --- | --- | --- | --- | --- | --- |
| 1 | *Q. verrucospinosa* (AUP00392) |  |  |  |  |  |  |  |  |  |  |  |  |  |  |  |  |  |  |  |  |  |
| 2 | *Q. verrucospinosa* (AUP00393) | 0 |  |  |  |  |  |  |  |  |  |  |  |  |  |  |  |  |  |  |  |  |
| 3 | *Q. verrucospinosa* (EU979805) | 0.2 | 0.2 |  |  |  |  |  |  |  |  |  |  |  |  |  |  |  |  |  |  |  |
| 4 | *Q. verrucospinosa* (DQ118482) | 0.2 | 0.2 | 0 |  |  |  |  |  |  |  |  |  |  |  |  |  |  |  |  |  |  |
| 5 | *Q. verrucospinosa* (EU979809) | 0.2 | 0.2 | 0 | 0 |  |  |  |  |  |  |  |  |  |  |  |  |  |  |  |  |  |
| 6 | *Q.* cf. *verrucospinosa* 1 (EU979813) | 5.1 | 5.1 | 4.6 | 4.6 | 4.9 |  |  |  |  |  |  |  |  |  |  |  |  |  |  |  |  |
| 7 | *Q.* cf. *verrucospinosa* 1 (EU979850) | 5.1 | 5.1 | 4.6 | 4.6 | 4.9 | 0 |  |  |  |  |  |  |  |  |  |  |  |  |  |  |  |
| 8 | *Q.* cf. *verrucospinosa* 3 (EU979804) | 5.3 | 5.3 | 5.5 | 5.5 | 5.5 | 6.4 | 6.4 |  |  |  |  |  |  |  |  |  |  |  |  |  |  |
| 9 | *Q.* cf. *verrucospinosa* 2 (EU979812) | 2.7 | 2.7 | 2.4 | 2.4 | 2.5 | 6.4 | 6.4 | 5.3 |  |  |  |  |  |  |  |  |  |  |  |  |  |
| 10 | *Q.* cf. *verrucospinosa* 3 (EU979803) | 5.3 | 5.3 | 5.5 | 5.5 | 5.5 | 6.4 | 6.4 | 0.5 | 5.8 |  |  |  |  |  |  |  |  |  |  |  |  |
| 11 | *Q. boulengeri* (DQ118477) | 5.6 | 5.6 | 5.3 | 5.3 | 5.4 | 5.6 | 5.6 | 6.0 | 5.6 | 6.0 |  |  |  |  |  |  |  |  |  |  |  |
| 12 | *Q. boulengeri* (EU979821) | 4.9 | 4.9 | 4.6 | 4.6 | 4.7 | 4.9 | 4.9 | 5.3 | 4.9 | 5.3 | 1.2 |  |  |  |  |  |  |  |  |  |  |
| 13 | *Q. exilispinosa* (DQ118484) | 5.6 | 5.6 | 5.5 | 5.5 | 5.3 | 6.4 | 6.4 | 6.4 | 5.3 | 6.4 | 5.8 | 6.0 |  |  |  |  |  |  |  |  |  |
| 14 | *Q. jiulongensis* (EU979801) | 4.9 | 4.9 | 4.2 | 4.2 | 4.7 | 5.8 | 5.8 | 4.5 | 4.1 | 4.5 | 4.7 | 4.0 | 3.4 |  |  |  |  |  |  |  |  |
| 15 | *Q. jiulongensis* (DQ118485) | 5.1 | 5.1 | 4.4 | 4.4 | 4.9 | 6.0 | 6.0 | 4.7 | 3.9 | 4.7 | 4.9 | 4.3 | 3.6 | 0.2 |  |  |  |  |  |  |  |
| 16 | *Q. shini* (DQ118486) | 7.1 | 7.1 | 6.7 | 6.9 | 6.9 | 7.8 | 7.8 | 7.7 | 6.4 | 7.5 | 7.4 | 6.2 | 7.7 | 6.8 | 7.1 |  |  |  |  |  |  |
| 17 | *Q. shini* (DQ118487) | 7.1 | 7.1 | 6.7 | 6.8 | 6.9 | 7.8 | 7.8 | 7.5 | 6.2 | 7.5 | 7.4 | 6.2 | 7.5 | 6.6 | 6.8 | 0.2 |  |  |  |  |  |
| 18 | *Q. spinosa* (DQ118481) | 6.1 | 6.1 | 5.9 | 5.9 | 5.9 | 3.4 | 3.4 | 7.0 | 6.6 | 7.0 | 5.4 | 4.7 | 6.8 | 5.9 | 6.1 | 8.6 | 8.5 |  |  |  |  |
| 19 | *Q. spinosa* (DQ118480) | 6.2 | 6.2 | 5.5 | 5.5 | 6.0 | 3.4 | 3.4 | 6.7 | 6.4 | 6.7 | 5.3 | 4.7 | 6.9 | 5.6 | 5.8 | 8.5 | 8.5 | 0.4 |  |  |  |
| 20 | *Q. exilispinosa* (EU979798) | 5.1 | 5.1 | 5.6 | 5.5 | 5.4 | 7.1 | 7.1 | 6.0 | 5.4 | 6.0 | 6.2 | 6.0 | 1.5 | 3.5 | 3.7 | 8.0 | 7.7 | 7.8 | 7.8 |  |  |
| 21 | Q. *exilispinosa* (EU979797) | 4.9 | 4.9 | 5.3 | 5.3 | 5.1 | 6.9 | 6.9 | 5.7 | 5.1 | 5.7 | 6.0 | 5.7 | 1.3 | 3.2 | 3.4 | 7.7 | 7.5 | 7.5 | 7.6 | 0.2 |  |
| 22 | *Q. yei* (DQ118488) | 5.0 | 5.0 | 4.9 | 4.8 | 4.8 | 6.4 | 6.4 | 5.9 | 4.8 | 6.3 | 6.4 | 5.7 | 5.7 | 5.2 | 5.5 | 6.6 | 6.3 | 7.1 | 6.9 | 5.7 | 5.4 |
