# Supplementary material for: First national record of Quasipaaverrucospinosa (Bourret, 1937) (Amphibia: Anura: Dicroglossidae) from Thailand with further comment on its taxonomic status
Source: Biodivers Data J. 2021 Sep 30;9:e70473. doi: 10.3897/BDJ.9.e70473 (PMC8497459; doi:10.3897/BDJ.9.e70473)
Supplement: Supplementary material 3 — Measurement (in mm) and proportions of the series of Quasipaaverrucospinosa in Thailand. (M= Male, F= Female; for other abbreviations see Materials and methods). [file bdj-09-e70473-s003.docx]

**Table 3.** Measurement (in mm) and proportions of the series of *Quasipaa verrucospinosa* in Thailand. (M= Male, F= Female; for other abbreviations see Materials and methods).

| Specimen | AUP  00531 | AUP  00532 | AUP  00533 | AUP  00534 | AUP  00392 | AUP  00393 | AUP  00530 | AUP  01609 |
| --- | --- | --- | --- | --- | --- | --- | --- | --- |
| SEX | M | M | M | M | F | F | F | F |
| SVL | 109.1 | 116.8 | 99.1 | 114.1 | 83.4 | 112.3 | 112.7 | 88.5 |
| HL | 37.2 | 41.9 | 34.8 | 40 | 29.4 | 38.5 | 38.3 | 32.1 |
| SL | 13.5 | 15 | 12.7 | 15.1 | 11.1 | 15 | 15.2 | 12.5 |
| EL | 12.5 | 13.7 | 12.6 | 12.9 | 9.1 | 14 | 13.2 | 11.3 |
| N–EL | 7.3 | 8 | 7.3 | 8.5 | 5.3 | 6.6 | 8.5 | 6.7 |
| HW | 46.6 | 52.5 | 41.8 | 48.2 | 34.8 | 48.3 | 47.9 | 37 |
| IND | 10.1 | 11.3 | 9 | 10.3 | 8.3 | 10 | 9.9 | 7.4 |
| IOD | 9.1 | 9.3 | 8.8 | 9.2 | 8 | 10 | 9.7 | 7.9 |
| UEW | 10.1 | 10.9 | 9.8 | 9.5 | 7.4 | 10.8 | 10.9 | 8.4 |
| FLL | 71.6 | 75.2 | 61.6 | 71.7 | 58.1 | 67.5 | 69.8 | 52.3 |
| LAL | 50.6 | 55.2 | 45.7 | 51.7 | 35.3 | 45 | 49.8 | 39.6 |
| HAL | 29.2 | 32.5 | 26.3 | 29.9 | 21.6 | 28.8 | 30.5 | 23.3 |
| 1FL | 12.2 | 13.7 | 11.6 | 13 | 8.2 | 11.6 | 13.3 | 9.8 |
| IPTL | 7.9 | 9.5 | 7.2 | 8.2 | 5.8 | 8.2 | 7.9 | 6.3 |
| OPTL | 7.5 | 6.9 | 6.3 | 8 | 5.3 | 7.3 | 7.4 | 5.1 |
| 3FDD | 2.8 | 3.1 | 2.2 | 3.1 | 2 | 2.8 | 3 | 2.6 |
| HLL | 192.3 | 211.2 | 177.4 | 201.3 | 141.3 | 185.7 | 193.1 | 151.9 |
| TL | 63.8 | 68.5 | 60.2 | 67.1 | 46.5 | 60.3 | 65.1 | 51.4 |
| FL | 79.9 | 86.2 | 71.9 | 82.3 | 58.7 | 77.2 | 76.2 | 61.8 |
| IMTL | 9.2 | 10.6 | 10.7 | 10.9 | 7.2 | 10.8 | 12.1 | 8.7 |
| 1TOEL | 14.3 | 16.3 | 14.1 | 15.4 | 11.8 | 14.5 | 15.1 | 12.7 |
| 4TDD | 3.9 | 4.1 | 3.8 | 3.8 | 2.9 | 4.5 | 4.1 | 3.2 |
| TD | 4.8 | 5.7 | 4.9 | 5.5 | 4.9 | 5.1 | 5.7 | 5 |
| OMTL | 5.8 | 6.5 | 5.6 | 5.4 | 5.5 | 7 | 6.8 | 6.7 |
